# Single-incision bilateral laparoscopic oophorectomy

**DOI:** 10.4103/0972-9941.72392

**Published:** 2011

**Authors:** Deepraj Bhandarkar, Avinash Katara, Vinay Deshmane, Gaurav Mittal, Tehemton E Udwadia

**Affiliations:** Division of Minimal Access Surgery, P D Hinduja National Hospital & Medical Research Centre, Veer Savarkar Marg, Mahim, Mumbai-400016, India; 1 Division of Surgical Oncology, P D Hinduja National Hospital & Medical Research Centre, Veer Savarkar Marg, Mahim, Mumbai-400016, India

**Keywords:** Laparoscopy, oophorectomy, single-incision, single port access

## Abstract

Although single-incision laparoscopic surgery made an appearance on the surgical scene only recently, it is being increasingly applied in the treatment of a variety of disorders. We report single-incision bilateral laparoscopic oophorectomy and salpingooophorectomy performed in two patients who had previously undergone breast conservation surgery for early breast cancer. Each procedure was undertaken using two 5-mm and one 3-mm ports inserted through a 2-cm transverse supraumbilical incision and standard laparoscopic instruments. The operative time was 50 and 65 min respectively and the blood loss negligible. The patients were discharged 36 and 24 h after surgery, required minimal postoperative analgesia and remain well at a follow up of 19 and 17 months, respectively. With the benefit of improved cosmesis, the single-incision approach holds the potential to replace the traditional bilateral laparoscopic oophorectomy.

## INTRODUCTION

In recent years surgeons’ quest to reduce the size and number of incisions used for minimal access surgery has lead to the development of several innovations. Natural orifice translumenal endoscopic surgery (NOTES) and single-incision laparoscopic surgery (SILS) have been two such advances. Because of the potential hazards of trans-visceral access associated with NOTES and lack of proper instrumentation for surgery through transvaginal access, SILS has generated a far greater interest. It seems to be gaining popularity among the surgeons and has already been used for performing cholecystectomy,[1] appendicectomy,[2] colon resection[3] and splenectomy.[4]

We report two patients with previously operated, hormone-dependent breast cancer who successfully underwent a single-incision bilateral laparoscopic oophorectomy as systemic therapy.

## CASE REPORTS

### Case 1

A 37-year-old woman was referred to us for a bilateral laparoscopic oophorectomy (BLO) 7 months after her breast conservation surgery and axillary clearance for a T2N1M0 breast cancer. The tumour was oestrogen and progesterone receptor positive and 2 out of 16 lymph nodes contained metastases. Postoperatively, she had received radiotherapy as well as chemotherapy with Paclitaxel (Taxol, Bristol-Myers Squibb, New York, USA). She had been commenced on Tamoxifen (Tamoxifen, Dabur, Ghaziabad, India) and Goserlin Acetate (Zoladex, Astra Zeneca, Bangalore, India). As the patient was keen to undergo ovarian ablation as a one-time therapy, she was counselled for and offered single-incision BLO.

The surgery was performed (by DB) with the patient in Trendlenberg position with both arms tucked by the side. The bladder was catheterized and general endotracheal anaesthesia was administered. A 2-cm supraumbilical transverse incision was deepened to the fascia. Dissection was carried out to expose the sheath for a distance of 1.5 cm on either side. The superior lip of the skin incision was retracted and a small incision was made on the sheath. The edges of the skin were elevated with towel clips and a 5-mm Endopath Xcel port (Ethicon Endosurgery, Guyanabo, Puerto Rico, USA) was guided into the peritoneal cavity. Insufflation with CO_2_ was begun and maintained at 12 mmHg. A 30° 5-mm laparoscope attached to an Image-1 high-definition endocamera (Karl Storz, Tuttlingen, Germany) was used for visualization. Additional 5-mm and 3-mm metal ports were placed adjacent to the first port through the same skin incision. Each ovary was grasped with a curved dissector and the pedicle was coagulated using an ultrasonic shears (Harmonic Scalpel, Ethicon Endosurgery, USA). The ovary was dissected free and hemostasis was confirmed. The fascial incisions were joined, a 12-mm Endopath Xcel port was introduced in the abdomen and the carboperitoneum re-established. The endocamera then shifted to a nephroscope (Karl Storz, Tuttlingen, Germany), which was passed through the 12-mm port. Each ovary was retrieved without contact with the wound using a long grasping forceps introduced through the working channel of the nephroscope. The fascial incision was closed with non-absorbable sutures and skin approximated with a subcuticular suture. The total operating time was 50 min.

The patient received parenteral analgesics for the first 24 h and was discharged 36 h after surgery. She did not require any oral analgesics. At 1 week follow up, the wound had healed well and the patient remains well 19 months after surgery. Histopathologically, both the ovaries were normal.

### Case 2

**Figure 1:**
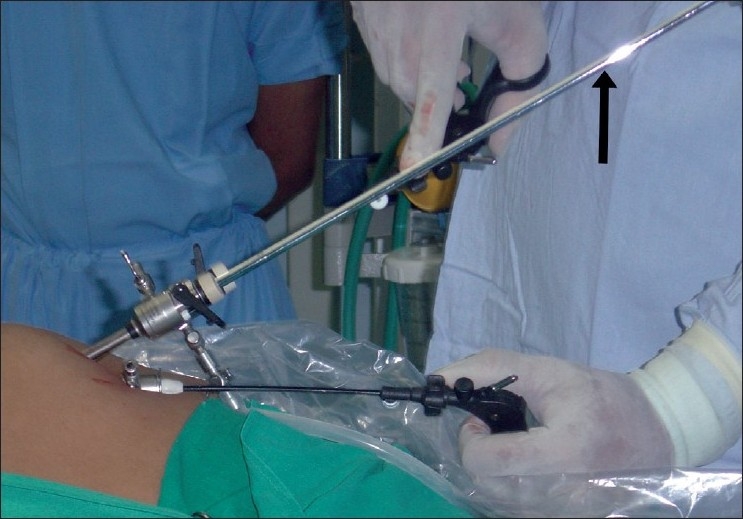
Configuration of ports

**Figure 2:**
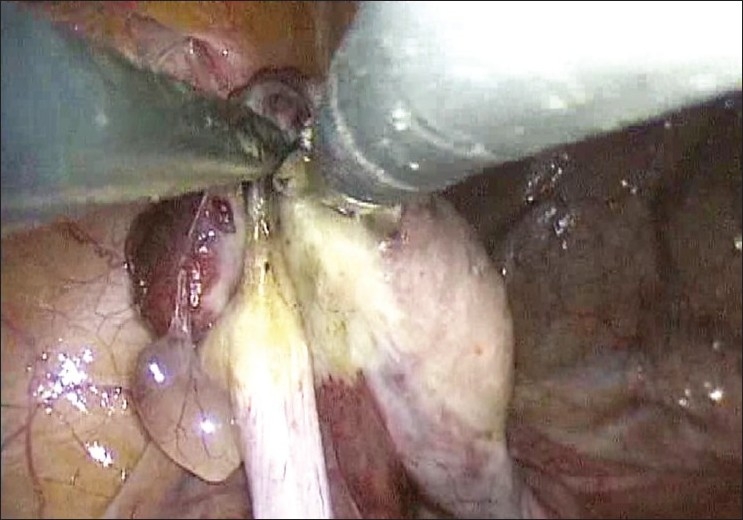
Dissection of left ovary and tube in progress

**Figure 3:**
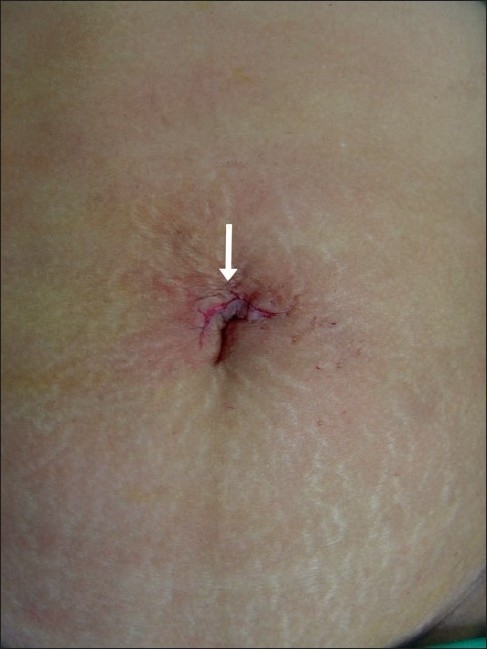
Scar after surgery

A 42-year-old lady who had previously undergone surgery for a T1N1M0 breast cancer underwent bilateral SILS salpingo-oophorectomy. The port configuration was identical to that used in the first patient [Figure 1]. In this patient we used a 51-cm, 5-mm, 30° bronchoscope (Karl Storz, Tuttlingen, Germany) as laparoscope. After preliminary laparoscopy, the ovarian vessels and the round ligaments on each side were coagulated using a bipolar energy source and the ovaries along with the tubes were excised [Figure 2]. One of the 5-mm trocar was exchanged for a 10-mm trocar, a plastic bag was introduced in the abdomen and the specimens were extracted after placing them in the bag. The fascia was closed with non-absorbable suture and skin with a subcuticular suture [Figure 3]. Total operative time was 65 min. The patient received parenteral analgesics for the first 24 h and was well enough to go home 24 h after surgery. She did not require any oral analgesics. At 1 week follow up, the wound had healed well and the patient remains well 17 months after surgery. Histopathologically, the left ovary was normal and the right one showed micrometastases from breast cancer.

## DISCUSSION

Ovarian ablation has been used for more than a century in the treatment of breast cancer. Potentially reversible ovarian suppression can be achieved by using luteinizing hormone releasing hormone (LHRH) analogues and irreversible ovarian failure can be accomplished by ovarian irradiation or surgical oophorectomy. In premenopausal women, the impact of ovarian ablation on the reduction in the risk for recurrent breast cancer was well established by the Oxford overview analysis.[5] This report showed that at 15 years of follow-up, premenopausal women treated with ovarian ablation had statistically significantly higher relapse-free survival as also overall survival rates. In women with a strong family history of breast and or ovarian cancer who are *BRCA1* or *BRCA2* mutation carriers, prophylactic bilateral salpingo-oophorectomy (PBSO) has been shown to significantly reduce the risk of ovarian, fallopian tubal and peritoneal cancer.[6] Furthermore, PBSO also provides a protective effect against breast cancer.[7]

Several authors have reported on the feasibility, safety and benefits of standard three-port BLO as a systemic therapy in patients with breast cancer. Mueller *et al*. in 1998 described 22 women undergoing BLO for metastatic breast cancer. Twelve of the 44 removed ovaries showed evidence of micrometastases. There were no intra- or postoperative complications. Kucera *et al*. reported on 90 women with early breast cancer undergoing laparoscopic bilateral salpingo-oophorectomy (BSO) either with or without hysterectomy.[9] Blood loss was minimal in both groups and the incidence of postoperative complications was low. Willsher *et al*. recently published a review of 70 patients undergoing BLO by breast surgeons with expertise in laparoscopic surgery.[10] Forty-three patients had the surgery as adjuvant endocrine treatment of early breast cancer, 13 for prophylaxis, 7 for endocrine and prophylactic reasons and 7 for treatment of metastatic breast cancer. There were no conversions or significant complications. History of prior abdominal surgery (in 29 patients) did not affect the success of surgery or its outcome.

Fadar *et al*. recently reported a series of SILS in gynaecology.[11] They undertook endometrial cancer staging, ovarian cancer staging, retroperitoneal pelvic lymph node dissection, extrafascial hysterectomy, BSO, ovarian cystectomy in a total of 13 patients. Nine were operated laparoscopically and 4 with robotic assistance. Surgeries were carried out through a single 2-3 cm umbilical incision with using either a SILS port (Covidien, Mansfield, MA, USA) or a Gelport (Applied Medical, Rancho Santa Margarita, CA, USA). There were no complications and majority of the patients did not require any postoperative narcotic analgesics.

As our patients underwent SILS without the use of specialized access devices, low-profile trocars or roticulating/angulating instruments we faced several technical challenges. Lack of triangulation resulted in “sword fighting” of the instruments. This was overcome by the surgeon deliberately slowing down the hand movements as also by restricting the movements to small arcs. In the first patient, during turning of the laparoscope there was clashing of the non-coaxial light cable. We used the zoom facility on the camera that allowed the tip of the laparoscope to be kept away from the operative field and yet provide a close-up view. In the second patient, using an extra-long telescope allowed the endocamera and the cable to move away from the operative field and made manipulation of instruments much easier. Mindful that a small percentage of patients with breast cancer harbour metastases in the ovaries, we extracted the specimens without contact with the incision. A nephroscope with a working channel, an instrument readily borrowed from the urologist colleagues, was used for the purpose in the first patient and a bag in the second. We strongly feel that performing single-incision surgery using standard instruments with the help of innovations can make it cost-effective. The learning curve inherent to SILS can be shortened if the surgery is performed by an experienced laparoscopic surgeon supported by a constant team.

SILS is a burgeoning technical advance whose advantages in terms of reduced pain, faster recovery and reduced convalescence are by no means established. More data are needed on surgical outcomes, equivalency or superiority over traditional laparoscopic procedures as well as the incidence of postoperative wound complications like seroma, infection and port-site herniation–likely resulting from increased trauma to the periumbilical tissue. However, if these issues are settled, single-incision BLO holds the potential to replace the traditional BLO due to the improved cosmesis it offers.
